# Critical inhaler technique errors in Swedish patients with COPD: a cross-sectional study analysing video-recorded demonstrations

**DOI:** 10.1038/s41533-021-00218-y

**Published:** 2021-02-09

**Authors:** Johanna Sulku, Kristina Bröms, Marieann Högman, Christer Janson, Karin Lisspers, Andrei Malinovschi, Håkan Melhus, Björn Ställberg, Elisabet I. Nielsen

**Affiliations:** 1 Department of Pharmacy, Uppsala University, Uppsala, Sweden; 2 Centre for Research and Development, Uppsala University/Region Gävleborg, Gävle, Sweden; 3 Department of Public Health and Caring Sciences, Family Medicine and Preventive Medicine, Uppsala University, Uppsala, Sweden; 4 Department of Medical Sciences, Respiratory, Allergy and Sleep Research, Uppsala University, Uppsala, Sweden; 5 Department of Medical Sciences, Clinical Physiology, Uppsala University, Uppsala, Sweden; 6 Department of Medical Sciences, Clinical Pharmacogenomics and Osteoporosis, Uppsala University, Uppsala, Sweden

**Keywords:** Chronic obstructive pulmonary disease, Drug regulation

## Abstract

A correct use of inhaler devices is essential in chronic obstructive pulmonary disease (COPD) treatment. Critical errors were studied by analysing 659 video-recorded demonstrations of inhaler technique from 364 COPD patients using six different inhaler device models. The majority of the included patients used two (55%) or more (20%) device models. Overall, 66% of the patients made ≥1 critical error with at least one device model. The corresponding numbers for patients using 1, 2 and ≥3 device models were 43%, 70% and 86%, respectively. The only factor associated with making ≥1 critical error was simultaneous use of two (adjusted odds ratios (aOR) 3.17, 95% confidence interval (95% CI) 1.81, 5.64) or three or more (aOR 8.97, 95% CI 3.93, 22.1) device models. In conclusion, the proportion of patients making critical errors in inhaler technique was substantial, particularly in those using several different device models. To obtain optimal COPD treatment, it is important to assess a patient’s inhaler technique and to minimise the number of inhaler device models.

## Introduction

Chronic obstructive pulmonary disease (COPD) is characterised by persistent respiratory symptoms and a high risk of exacerbations^1^. The preventive and maintenance pharmacological treatment is primarily administered through handheld inhaler devices^2^. There are several different inhaler device models on the market, with each model requiring a specific procedure for the optimal leverage of the inhaled drug^3–5^. Breezhaler and Handihaler are examples of single-dose dry-powder inhalers (sDPIs), where each drug dose is packed in a single-dose capsule^4^. Diskus (Accuhaler), Easyhaler, Genuair, Novolizer and Turbuhaler are examples of multi-dose DPIs (mDPIs), where the drug is often blended with carrier particles, and packed either in individual blister strips (Diskus (Accuhaler)) or in a powder reservoir (Easyhaler, Genuair, Novolizer and Turbuhaler)^4^. Pressurised metered-dose inhalers (pMDIs) are aerosol formulations where the drug is either dissolved or suspended in propellant gas^4,5^. The breath-actuated pMDI is an aerosol formulation where the release of the drug dose is triggered by the patient’s inhalation flow through the device^4^. The soft-mist inhaler (SMI) Respimat is another aerosol formulation, which generates a propellant-free inhalable cloud^4,5^.

The effect of inhalation therapy is highly dependent on the delivery of the inhaled drug to the lungs and mastering a correct inhaler technique is essential for effective treatment^6–8^. In particular, critical errors, that is, actions or inactions during the inhalation procedure that result in little or no drug being inhaled or reaching the lungs, should be avoided^6,7^. The types of critical errors that might occur vary between device models, as the inhaler handling and inhaler technique are different for each model. Coordination of actuation and inhalation is not needed with any of the DPI models or the breath-actuated pMDI^3^, but is important when inhaling a dose from the Respimat or the other pMDIs. The inspiratory flow rate required varies between inhaler device models. In general, the inhalation should be hard and fast for the DPI device models, and long and slow for the Respimat and the pMDIs^4,5^. As the DPIs are sensitive to moisture, breathing through the inhaler device might decrease the effective drug dose. Due to these differences, the inhaler device model or models should be chosen to suit each patient’s circumstances and abilities^4,9^. However, errors in inhaler technique remain a large problem in COPD care^10,11^.

Combining multiple drugs^12^ and inhaler devices^13^ is common in the treatment of COPD. Several inhaler devices are available with varying contents, of which some are available as fixed combinations^4,9^. However, detailed guidance is lacking regarding how to combine the different inhaler device models when more than one inhaler device is needed in a treatment regimen. Currently, there are limited data about critical inhaler technique errors in patients with COPD using multiple inhaler device models^14–16^, and previous studies have typically been restricted to studying a specific type and/or number of device models^14–18^. The aim of this study was to determine the prevalence and types of critical errors in inhaler technique among patients with COPD, and to investigate factors associated with critical errors.

## Results

**Fig. 1:**
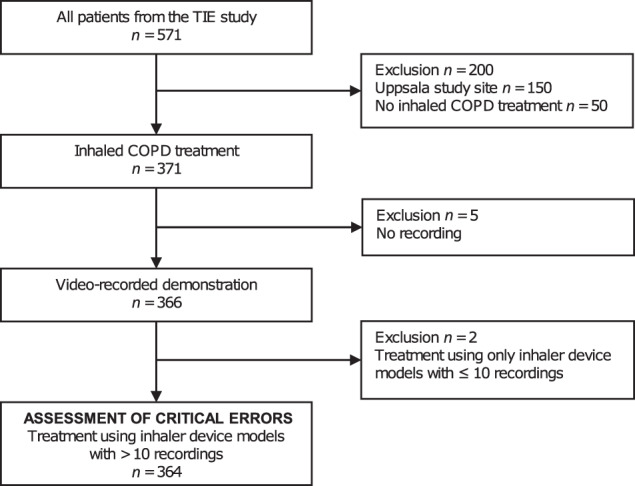
Flowchart of patients included in and excluded from the assessment of critical errors in inhaler technique. COPD chronic obstructive pulmonary disease, TIE tools for identifying exacerbations.

Of 371 eligible patients with inhaled COPD treatment, 364 were included in the assessment of critical errors in inhaler technique. Five patients were excluded due to missing recordings for all of the patient’s inhaler device models and two patients due to the use of only inhaler device models with ≤10 recordings (Fig. 1). Demographics and study characteristics of the patients included in the assessment of critical errors, and in the subgroups of patients making ≥1 critical error (*n* = 242; 66%), and no critical errors (*n* = 122; 34%) with at least one inhaler device, are presented in Table 1. Patient characteristics were similar in both subgroups, except for frequent exacerbations, secondary care contacts and simultaneous use of two or more inhaler device models, which were more frequent in patients with ≥1 critical errors.

**Table 1:** Demographics and study characteristics of the patients included in the assessment of critical errors, and for the subgroups of patients making ≥1 critical error and patients making no critical errors with at least one device model.

Variable	All (*n* = 364)	≥1 critical error (*n* = 242)	No critical errors (*n* = 122)	*P* value^a^
Sex				0.42
Female	212 (58%)	145 (60%)	67 (55%)	
Age (years)				
Mean (SD)	69 (8)	70 (8)	69 (8)	0.37
<65	84 (23%)	52 (21%)	32 (26%)	0.44
65–75	203 (56%)	135 (56%)	68 (56%)	
>75	77 (21%)	55 (23%)	22 (18%)	
BMI (kg/m^2^)				0.53
<22	48 (13%)	29 (12%)	19 (16%)	
22–30	217 (60%)	143 (60%)	74 (61%)	
>30	96 (27%)	67 (28%)	29 (23%)	
Current smoker	98 (27%)	69 (29%)	29 (24%)	0.40
Education				0.95
Elementary school	224 (62%)	148 (61%)	76 (62%)	
Upper secondary school	95 (26%)	64 (27%)	31 (25%)	
University	44 (12%)	30 (12%)	14 (11%)	
Severity of airflow limitation				
FEV_1_ % predicted mean (SD)	54 (17)	53 (17)	56 (17)	0.09
FEV_1_ ≥ 80%	24 (7%)	14 (6%)	10 (8%)	0.18
50% ≤ FEV_1_ < 80%	198 (54%)	132 (55%)	66 (54%)	
30% ≤ FEV_1_ < 50%	105 (29%)	66 (27%)	39 (32%)	
FEV_1_ < 30%	37 (10%)	30 (12%)	7 (6%)	
CAT score ≥ 10	227 (62%)	158 (65%)	69 (57%)	0.13
mMRC score ≥ 2	172 (47%)	117 (48%)	55 (45%)	0.63
Frequent exacerbations	76 (21%)	60 (25%)	16 (13%)	0.01
Care level				0.01
Primary care	324 (89%)	208 (86%)	116 (95%)	
Secondary care	40 (11%)	34 (14%)	6 (5%)	
Visit to physician in preceding year	150 (41%)	108 (45%)	42 (34%)	0.07
Visit to asthma/COPD nurse in preceding year	255 (70%)	168 (70%)	87 (71%)	0.89
Number of device models used				<0.0001
1 device model	91 (25%)	39 (16%)	52 (43%)	
2 device models	199 (55%)	139 (57%)	60 (49%)	
≥3 device models	74 (20%)	64 (27%)	10 (8%)	

Data are presented as number (%) of patients, unless otherwise indicated.

*BMI* body mass index, *CAT* COPD assessment test, *COPD* chronic obstructive pulmonary disease, *FEV*_*1*_ forced expiratory volume in 1 s, *frequent exacerbations* ≥2 exacerbations treated in primary care or at an emergency department and/or ≥1 hospital admissions during the preceding year due to worsening in COPD, *mMRC* Modified Medical Research Council Dyspnoea Scale, *SD* standard deviation.

^a^Comparison of variables between the subgroups were tested with Pearson’s *χ*^2^ test or Fisher’s exact test for categorical variables and with unpaired *t* test for continuous variables.

### Inhaler device models

**Fig. 2:**
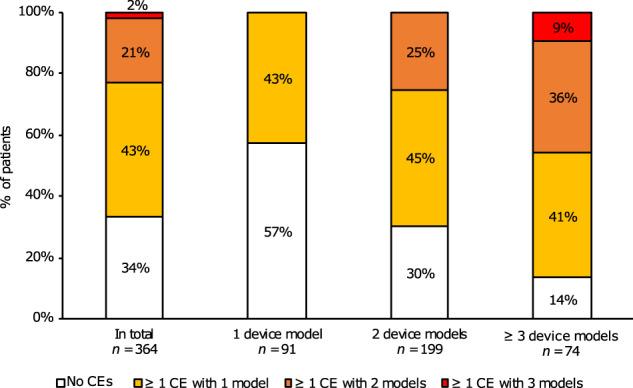
Critical inhaler technique errors as percentages of COPD patients making ≥1 critical error with one, two or three or more device models, in total and by number of device models used. CE critical error.

Overall, Turbuhaler, Handihaler and Easyhaler were the most commonly used inhaler device models with 227, 181 and 84 recordings available, respectively (Table 2). Most of the patients had COPD treatment delivered from a combination of two different inhaler device models (*n* = 199), but patients combining up to four different inhaler device models were identified (Fig. 2 and Supplementary Table 7). Among patients using a single inhaler device model, Turbuhaler (*n* = 33) was the most commonly used model. The most frequently used combinations of two device models were Handihaler + Turbuhaler (*n* = 90), Genuair/Novolizer + Turbuhaler (*n* = 24), Respimat + Turbuhaler (*n* = 16) and Easyhaler + Handihaler (*n* = 15). Diskus (Accuhaler) + Handihaler + Turbuhaler (*n* = 17) and Easyhaler + Handihaler + Turbuhaler (*n* = 11) were the most commonly used combinations of three device models (Supplementary Table 7).

**Table 2:** Number (%) of recordings with critical errors (CEs) and types of errors in total and per inhaler device model.

	In total	Breezhaler and/or Handihaler	Diskus (Accuhaler)	Easyhaler	Genuair and/or Novolizer	Turbuhaler	Respimat
*n* Recordings	659	188^a^	42	84	67^b^	227	51
No error	64 (10%)	13 (7%)	1 (2%)	4 (5%)	5 (7%)	33 (15%)	8 (16%)
≥1 CE	333 (51%)	68 (36%)	38 (90%)	47 (56%)	35 (52%)	125 (55%)	20 (39%)
≥1 non-CE only	262 (40%)	107 (57%)	3 (7%)	33 (39%)	27 (40%)	69 (30%)	23 (45%)
Number of CEs							
1 CE	263 (40%)	46 (24%)	31 (74%)	42 (50%)	31 (46%)	106 (47%)	7 (14%)
2 CEs	63 (10%)	18 (10%)	7 (17%)	5 (6%)	4 (6%)	18 (8%)	10 (20%)
3 CEs	7 (1%)	4 (2%)	0	0	0	0	3 (6%)
4 CEs	0	0	0	0	0	1 (0.4%)	0
Error types							
Not opening the device correctly^c^	16 (2%)	1 (0.5%)	0	0	1 (1%)	2 (1%)	12 (24%)
Errors in dose preparation and loading^c^	280 (42%)	39 (21%)	38 (90%)	44 (52%)	34 (51%)	110 (48%)	15 (29%)
No exhalation before inhalation	429 (65%)	124 (66%)	26 (62%)	55 (65%)	42 (63%)	154 (68%)	28 (55%)
Exhalation into the device (DPIs only)^c^	58 (9%)	23 (12%)	4 (10%)	7 (8%)	0	24 (11%)	NA
Not inhaling through mouth^c^	1 (0.2%)	0	0	0	0	0	1 (2%)
Errors in inhalation manoeuvre^c^	57 (9%)	31 (16%)	3 (7%)	1 (1%)	4 (6%)	10 (4%)	8 (16%)
No breath-holding after inhalation	321 (49%)	155 (82%)	31 (74%)	59 (70%)	44 (66%)		32 (63%)
Not resuming normal breathing after inhalation	9 (1%)	4 (2%)	0	2 (2%)	1 (1%)	2 (1%)	0
Empty capsule not removed	52 (8%)	52 (28%)	NA	NA	NA	NA	NA
The device is not closed	93 (14%)	24 (13%)	9 (21%)	20 (24%)	10 (15%)	26 (11%)	4 (8%)

*DPI* dry-powder inhaler device, *NA* not applicable.

^a^Breezhaler *n* = 7, Handihaler *n* = 179, both *n* = 2.

^b^Genuair *n* = 40, Novolizer *n* = 23, both *n* = 4.

^c^A critical error.

### Critical errors in inhaler technique

The prevalence of critical and non-critical errors per inhaler device model are presented in Table 2. The 364 patients included in the assessment performed a total of 659 demonstrations of inhaler technique. One or more critical error in inhaler technique was identified in 51% of the recordings, varying between 36 and 90% depending on the device model. The majority of the identified critical errors, regardless of the inhaler device model, were related to dose preparation and loading (21–90%). Other common critical errors were exhalation into the device prior to inhalation for the DPIs (0–12%), errors in inhalation manoeuvre (1–16%) and errors related to opening the device for the Respimat (24%) (Table 2). In total, 242 patients (66%) made one or more critical error (Fig. 2). Among the users of 1, 2 and ≥3 inhaler device models, ≥1 critical error was made by 43%, 70% and 86%, respectively. As might be expected, the percentage of patients with no critical errors decreased as the number of combined inhaler device models increased (Fig. 2). For patients using two inhaler device models, 45% made ≥1 critical error with one of the device models, and 25% made errors with both models. For patients with three or more device models, 41%, 36% and 9% made ≥1 critical error for 1, 2 and 3 models, respectively.

### Factors associated with critical errors

Lower lung function, frequent exacerbations, secondary care contact, planned visits to a physician and simultaneous use of two or three or more inhaler device models were identified as statistically significant factors for making ≥1 critical error in an unadjusted logistic regression analysis (Supplementary Table 8). Use of a combination of two or three or more inhaler device models was the only statistically significant independent factor remaining in an adjusted logistic regression analysis (Table 3). The odds of making ≥1 critical error was three times and nearly nine times higher in patients using a combination of two or three or more inhaler device models, respectively, in comparison with patients using only one device model.

**Table 3:** aORs with 95% CIs for variables associated with ≥1 critical error with at least one of a patient’s inhaler device models in a multivariable logistic regression analysis.

Variable	aOR^a^ (95% CI)
Sex	
Male	1
Female	1.20 (0.74, 1.94)
Age (years)	
<65	1
65–75	1.42 (0.80, 2.51)
>75	1.78 (0.87, 3.72)
Lung function	
FEV_1_ ≥ 80%	1
50% ≤ FEV_1_ < 80%	1.09 (0.43, 2.70)
30% ≤ FEV_1_ < 50%	0.53 (0.19, 1.42)
FEV_1_ < 30%	1.05 (0.28, 4.04)
Frequent exacerbations	
No	1
Yes	1.24 (0.64, 2.51)
Care level	
Primary care	1
Secondary care	1.91 (0.69, 6.01)
Visit to physician	
No	1
Yes	1.10 (0.66, 1.84)
Number of device models used	
1 device model	1
2 device models	3.17 (1.81, 5.64)
≥3 device models	8.97 (3.93, 22.1)

*aOR* adjusted odds ratios, *CI* confidence interval, *FEV*_*1*_ forced expiratory volume in 1 s, *frequent exacerbations* ≥2 exacerbations treated in primary care or at emergency department and/or ≥1 hospital admissions during the preceding year due to worsening in chronic obstructive pulmonary disease.

^a^Adjusted for all variables.

## Discussion

This cross-sectional study in primary and secondary care patients with COPD demonstrates that critical errors in inhaler technique are frequent, as two-thirds of the patients made one or more critical error. In addition, the use of several inhaler device models increased the odds of critical errors; an important finding, since as many as 75% of the patients in the study population combined two or more different inhaler devices.

In this study, one or more critical error was made by 66% of the COPD patients with at least one inhaler device model they were currently prescribed and using. Corresponding numbers in previous studies including solely COPD outpatients range between 25 and 78%^15–20^. There are some methodological differences between studies that might explain some of these discrepancies. Here, data were collected through video recordings, a method previously shown to be reliable in the assessment of patients’ inhaler technique^21^. The researchers could watch the recordings as many times as needed, which might have improved the assessment. The final assessments of critical errors for every statement in the checklists, and for each patient, were based on a consensus between two observers, a clinical pharmacist and an asthma/COPD nurse, both experienced in assessing patients’ inhaler technique. Similarly, in the study by Dabrowska et al.^20^ also showing a high critical error rate (78%), the inhaler technique demonstrations were assessed by two observers, a pulmonologist and a trained medical student, reaching a consensus in each assessment. In other studies, showing lower critical error rates, the data were collected and analysed either by unspecified health care personnel^15^, 212 general practitioners or 50 pulmonologists^17^, or a single registered nurse^18^ or pulmonologist^19^, based on single non-recorded episodes of inhaler technique demonstration.

Further, patients using more than one device model, regardless of if it was prescribed as needed or as a regular treatment, demonstrated the inhaler technique separately for each device model. In the end, the device models Ellipta and Spiromax and the pMDIs were excluded from the analysis due to too few observations. As previous studies have often had pre-specified restrictions in the type and/or number of device models to be included^14–18,22^, we believe the results presented here may better reflect real-life use of inhalers in the treatment of COPD.

As there are currently no validated tools for the assessment of critical errors in inhaler technique, there are differences in the definition of critical errors between studies. The inhalation manoeuvre, that is, inhalation flow rate and duration, is a potential source of critical errors. The checklist used in this study for each device model included a step evaluating if the inhalation manoeuvre was demonstrated as described in the patient information leaflets, for example, hard and fast for DPIs and slow and steady for the Respimat, as recommended^23^, and performed in the previous studies^15,16,18,19,22^. Thus, the inhalation manoeuvre was evaluated visually, using a similar procedure as in usual primary care, or at respiratory wards or outpatient clinics. However, no training equipment, for example, In-Check DIAL^24^ and Flo-tone (Clement Clarke International Ltd, https://www.haag-streit.com/clement-clarke/products/inhaler-technique/flo-tone-trainer/), or other trainer whistles or inhalation trainers were used to confirm the inspiratory flow. Further, even though exhalation through the DPI mouthpiece before inhalation significantly lowers the delivered drug dose^25^, this was not included as a potential critical error in all previous studies^16,18,19,22^. On the other hand, according to the criteria used in this study, no dose remaining on the dose counter for the mDPIs^17^ and lack of cartridge in the device for Respimat^17^ were not included as critical errors, as this could not be assessed properly for the demo inhalers. Further, powder remaining in the capsule at the end of inhalation was not included as a separate critical error for the sDPIs, as this step was included in the assessment of the inhalation manoeuvre. Thus, we assumed that there was no powder left in the capsule if the inhalation manoeuvre was performed as described in the patient information leaflet, that is, the capsule was rattling and no remaining powder was left in the capsule by the end of the inhalation.

In this study including only patients with COPD, the simultaneous use of multiple device models was the only factor significantly associated with critical errors, as also found in previous studies including patients with COPD^15,16^, and asthma and COPD^22^. In an observational study by Wieshammer and Dreyhaupt^26^, age was identified to be associated with critical errors. However, they used a slightly more heterogeneous study population with a wider age range and included patients on inhaler device treatment irrespective of diagnosis. Results from previous observational studies including only patients with COPD^14–16,19^ and investigating association of critical errors and simultaneous use of multiple device models are conflicting. van der Palen et al.^14^ showed in a randomised study with a cross-over design, including COPD patients naive to the device models studied, that patients treated with a single fixed-dose Ellipta DPI made fewer critical errors compared with patients using a combination of Diskus and Handihaler or Turbuhaler and Handihaler. Collier et al.^15^ compared single device model treatment with Ellipta against different dual DPI model treatment combinations, and found that the odds of making ≥1 critical error were up to seven times higher in patients using a combination of two inhaler device models, compared with patients using a single Ellipta inhaler. Khassawneh et al.^16^ showed, also in a cross-sectional study including patients using device models Diskus, Turbuhaler and pMDI, that those who were using more than one device model more often made critical errors in inhaler technique. However, other observational studies in COPD patients^19^ have not identified this positive association between the use of multiple inhaler device models and critical errors.

As mentioned before, due to the lack of an established definition of critical errors, there are differences between studies and the error rates identified in different studies should be compared with that in mind. In our study, the most common type of critical error, irrespective of the device model, was related to dose preparation and loading, as also seen in the previous studies^15,16,18,19^. Depending on the device model, 21–90% of the recordings revealed such errors as compared to 0–50% in other studies^14–19^. Our higher numbers might be related to differences in the device models studied, data collection and/or analysis methods, as explained earlier. The highest error rate related to dose preparation and loading was found in patients using Diskus. However, our definition for a correct dose preparation and loading with Diskus, “the patient holds the device with the mouthpiece toward him-/herself and slides the lever only once, until a click can be heard. The patient does not shake or turn the inhaler device upside down after sliding the lever”, might have been broader compared to the definitions used in previous studies, for example, “lever is not pushed back, and shook the inhaler after dose preparation”^15,27^, “failure to slide the lever until it clicks, or not keeping inhaler horizontally”^19^ or “failure to push the lever back fully until the “click” sound is heard”^18^.

Errors related to the inhalation manoeuvre were defined as critical for all device models included in this study. The identified error rates were somewhat lower compared with previous studies in patients with COPD, 1–16 vs. 0-29%^15,16,18,19^, depending on the device model. The wording for the definitions for incorrect inhalation manoeuvre varied in the previous studies^15,16,18^, but the message being the same, that is, the inhalation manoeuvre should be as fast and as long as possible for the DPIs, and slow and steady for the SMI. Noteworthy is that two previous studies^14,15^ categorised inhalation manoeuvre errors as non-critical, while another study^19^ defined incorrect inhalation manoeuvre as “incorrect inhalation were considered critical errors” with no further explanation. In a study by Molimard et al.^17^, errors were assessed that could reflect inhalation manoeuvre, and might be compared to our inhalation manoeuvre error rates, that is, remaining powder in the Breezhaler or Handihaler capsule by the end of inhalation (4–13^17^ vs. 16%) and lack of hand-lung synchronisation with the Respimat leading to smoke emanation (39^17^ vs. 16%). Similarly, another study^14^ identified that the capsule did not rattle in the chamber for 48% of the Handihaler users, which was over two times higher when compared to our inhalation manoeuvre error rate of 16% for Breezhaler/Handihaler.

A strength of this study is that it is based on data from an observational multicentre study including COPD patients from both primary and secondary care. In addition, a medication reconciliation was performed at each patient’s study visit, to ensure accurate and complete information about all the patients’ current inhaler devices. Further, the use of video-recorded material is a strength, as it allowed us to identify all the critical errors, since we could watch the recordings as many times as needed. This study also has some limitations. We included the six most common inhaler device models in the studied cohort of Swedish primary and secondary care patients with COPD, but left out inhaler device models with few observations, for example, pMDIs, which are more common in other parts of the world. The lack of standardised methods for the assessment of critical and non-critical errors in inhaler technique is also a weakness, which makes it difficult to compare results between studies. The inhalation manoeuvre error rates might be higher for device models where an objective measurement was included in the error criteria, that is, capsule rattling and a control for remaining powder in the capsule by the end of inhalation for the Breezhaler and the Handihaler, smoke emanation in the Respimat and the change in the colour in the control window with an audible click for the Genuair and the Novolizer. An identified weakness is also that 21 patients lacked recordings for one or more inhaler device model that they were using at the time. Only six of these 21 patients demonstrated the use of their included inhaler device models without critical errors, implying that the critical error rate could be even higher.

In conclusion, the results from this cross-sectional study in primary and secondary care patients with COPD showed that the proportion of patients making critical errors in inhaler technique was substantial. The most common critical error type was in dose preparation and loading. The majority of patients used multiple device models, which was associated with critical errors. As critical inhaler technique errors can lead to suboptimal COPD treatment, this study highlights the importance of assessing patients’ inhaler technique, choosing an optimal inhaler device model or combination of models and teaching the correct inhaler technique individually to each patient, in order to reduce critical errors in inhaler technique. Further, the number of inhaler device models combined should be minimised in patients for whom more than one drug is needed in the treatment of COPD. We propose using fixed-dose inhalers or adding a second device of the same inhaler device model that a patient has already mastered when a fixed-dose inhaler is not an option.

## Methods

### Study design and participants

The patients included in this study were part of the Tools for Identifying Exacerbations (TIE) study^28^. The TIE study is a prospective, cross-sectional, multicentre, observational study including Swedish primary and secondary care patients, with a spirometry-verified COPD diagnosis, age ≥40 years and ability to answer the study questionnaires. It is performed in the regions of Uppsala, Dalarna and Gävleborg. An exclusion criterion was a history of severe comorbidity, for example, metastasised cancer, severe heart failure or severe angina pectoris, which was clinically assessed at the inclusion visit^28^. Data were collected during 2014–2016 by research nurses at the study inclusion visits through spirometry, questionnaires and by video recordings of patients’ inhaler technique (performed in the regions of Dalarna and Gävleborg). Retrospective data were collected from electronic patient records. Of the patients included in the TIE study, those with ongoing inhaled COPD treatment and a video-recorded demonstration of inhaler technique for at least one of their inhaler device models were included in this assessment of critical errors (Fig. 1). Device models with less than ten video recordings were excluded.

### Lung function

Spirometry (Spiro Perfect Spirometer from Welch Allyn, Skaneateles Falls, NY, USA or Spirare Spirometer from Diagnostica AS, Oslo, Norway) was used to confirm COPD diagnosis, that is, airflow obstruction (a post-bronchodilator forced expiratory volume in 1 s (FEV_1_) divided by the highest value of either forced vital capacity (FVC) or slow vital capacity (SVC), thus FEV_1_/(FVC or SVC) < 0.70). The severity of lung function impairment was measured in a post-bronchodilator spirometry after a dose of 400 µg salbutamol without pre-test. Based on the severity of airflow limitation, using predicted FEV_1_, the patients were categorised as FEV_1_ > 80% (mild), FEV_1_ 50–80% (moderate), FEV_1_ 30–50% (severe) and FEV_1_ < 30% (very severe), in accordance with GOLD^9^.

### Body mass index

Body mass index (BMI) was calculated based on a patient’s weight and height, as measured at the inclusion visit. Based on BMI, the patients were categorised as underweight (BMI < 22 kg/m^2^), normal weight (BMI 22–30 kg/m^2^) or overweight (BMI > 30 kg/m^2^)^29^.

### Questionnaire

Patient-reported data were obtained through a questionnaire gathering information about age, sex, smoking status, educational level, COPD-related symptoms and planned visits with health care personnel due to COPD during the preceding year. Patients were categorised into groups based on age (<65 years, 65–75 years and >75 years), current smoking (yes/no), educational level (elementary school, upper secondary school or university), history of planned visits with a physician within the preceding year (yes/no) and history of planned visits with an asthma/COPD nurse within the preceding year (yes/no). Answers “don’t know” regarding planned visits were included in “no” (physician *n* = 4, asthma/COPD nurse *n* = 6). The COPD Assessment Test (CAT)^30^ and the Modified Medical Research Counsel Dyspnoea Scale (mMRC)^31,32^ were used to assess health status and symptoms. Patients with scores of ≥10 for CAT or ≥2 for mMRC were interpreted as having a high level of symptoms^2^.

### History of COPD exacerbations

Information about the level of care and history of COPD exacerbations during the 12 months preceding study inclusion was extracted retrospectively from electronic patient records. Exacerbations were defined as visits or care contacts that were unplanned according to the patient’s care plan, with increased respiratory symptoms requiring acute treatment with bronchodilators in a health care institution, and/or oral corticosteroids, and/or antibiotics, and/or admission to the emergency department and/or hospitalisation due to COPD. The definition of frequent exacerbations was ≥2 exacerbations treated in primary care or at an emergency department with a minimum of 14 days between the exacerbations, or ≥1 hospital admission due to worsening of COPD symptoms. Patients who had visited the respiratory clinic due to COPD during the year before the study entry were classified as having a secondary health care contact. Otherwise, the patient was considered as belonging to primary care.

### Assessment of critical errors in inhaler technique

Information about each patient’s current inhaler device model(s) was obtained in a medication reconciliation^33^ at the study visit^12^. The medication list brought by the patient and the list in the electronic patient records were reviewed by the research nurse or clinical pharmacist and the patient together. Patients were video recorded (Canon PowerShot SX600 HS camera from Canon Inc., NY, USA) when they demonstrated how they prepared and inhaled a dose from each of their prescribed inhaler device models. Nebulisers were not included in the review of inhaler technique. If possible, the patients’ own inhalers were used, otherwise empty demo inhalers with disposable mouthpieces were provided.

Critical and non-critical errors in inhaler technique were assessed using predefined and device-specific checklists (Supplementary Tables 1–6). The checklists were developed based on the patient information leaflets and previous studies^16,17,22^, and consisted of 8–10 statements, depending on the inhaler device model, which together described the procedure for a correct inhaler technique. The options for each statement were “agree”, “disagree” or “not visible in the video recording”, with a possibility to comment in free text. The option “agree” was to be used when no error was identified and “disagree” when the patient made an error. Further, a critical error was defined as an action or inaction during the inhalation procedure that in itself would have a detrimental impact on the delivery of the drug to the lung, as proposed by Usmani et al.^6^ Thus, errors identified from the inhalation demonstration in opening the device, dose preparation and loading, exhalation into the device prior to inhalation (DPIs only), not inhaling through the mouth or inhalation manoeuvre were categorised as critical because they could potentially decrease the expected effect^7,17,25^. Other errors identified were categorised as non-critical. The full versions of the checklists, including the categorisation of critical or non-critical errors, are provided in Supplementary Tables 1–6. Examples of observed critical errors in dose preparation and loading are shown in Table 4. In the assessment of critical errors, patients using inhaler device models requiring similar handling and inhaler techniques were grouped, that is, the single-dose DPIs (Breezhaler and Handihaler) and two of the multi-dose DPIs (Genuair and Novolizer). Other multi-dose DPIs (Easyhaler, Diskus and Turbuhaler) and the soft-mist inhaler Respimat were not grouped with other devices.

**Table 4:** Examples of possible critical errors performed during dose preparation and loading for device models included in the study.

Device model	Examples of possible critical errors
Breezhaler/Handihaler	• No capsule was inserted into the device • The patient pressed the side button(s) more than once
Diskus (Accuhaler)	• The device was not held with the mouthpiece toward the patient • The lever was slid more than once • The device was turned upside down after sliding the lever
Easyhaler	• The device was not shaken before loading • The device was not held in an upright position after being shaken • The button on top of the device was not pressed down until a click was heard • The button was pressed more than once • The device was shaken after the button was pressed
Genuair/Novolizer	• The device was not held horizontally when loading the dose • No click was heard when the button was pressed down
Respimat	• The cap was not held closed during loading • The device was not loaded by turning the clear base
Turbuhaler	• The device was not held upright (>45° from the vertical axis) during loading • No click was heard when the device was loaded by turning the coloured grip • The grip was turned more than once

Each video recording was examined for number and type of critical errors, one device model at a time. This was done separately by two investigators, a clinical pharmacist and an asthma/COPD nurse with experience of optimising patients’ inhaler technique. Before the evaluation, the investigators carefully reviewed the instructions in each patient information leaflet and any instruction films (Medicininstruktioner Sverige AB, https://www.medicininstruktioner.se/) provided by the manufacturer showing a correct inhaler technique. The final judgement of each patient’s inhaler technique was based on a consensus between the two investigators.

### Statistical analysis

Categorical variables were reported as frequencies and percentages, and continuous variables were presented as means and standard deviations. There were missing data regarding BMI (*n* = 3), education (*n* = 1) and planned visits to physician (*n* = 2), and to asthma/COPD nurse (*n* = 2). Twenty patients lacked recordings for one of their inhaler device models, and one patient lacked recordings for two models. The assessment of critical errors in these patients was based on their existing recordings (Supplementary Table 7). In total, the number of missing recordings per device was as follows: Diskus (Accuhaler) (*n* = 5), Easyhaler (*n* = 4), Handihaler (*n* = 3) and Turbuhaler (*n* = 10). Pearson’s *χ*^2^ test (or Fisher’s exact test, when applicable) was used for comparison of categorical variables between the subgroups of patients with ≥1 critical error and patients with no critical errors with at least one of the inhaler device models used. Comparison of means of continuous variables was performed using the unpaired *t* test. Each variable’s impact on critical errors in inhaler technique was compared between the groups making ≥1 critical error and those making no critical errors, using simple (unadjusted) and multivariable (adjusted) logistic regression analysis and presented in odds ratios. The factors potentially affecting critical errors in inhaler technique included in the unadjusted analysis were: sex, age, BMI, smoking status, educational level, lung function, symptom burden based on CAT and mMRC, frequent exacerbations, care level, planned visits to physician and asthma/COPD nurse due to COPD in the preceding year, and the number of device models used. Factors shown as statistically significant in the unadjusted analysis, as well as age and sex, were included in an adjusted model, in order to evaluate the independent effects of the factors. Two-sided tests were applied and a *p* value < 0.05 was considered statistically significant in all the analysis except when choosing variables in the adjusted logistic regression analyses, where a *p* value <0.1 was applied. Data management and statistical analyses were performed using R, version 3.4.0 (R Core Team, R Foundation for Statistical Computing, Vienna, Austria, 2017).

### Ethics

Ethical approval for the study was obtained from the Regional Review Board in Uppsala, Sweden (Dnr 2013/358), with an amendment specifying that video recording of the inhaler technique was approved (Dnr 2013/358/1). Written informed consent was obtained from all patients prior to study inclusion.

### Reporting summary

Further information on research design is available in the Nature Research Reporting Summary linked to this article.

## Supplementary information

Supplemental material

Reporting summary

## Data Availability

Data cannot be made freely available as they are subject to secrecy in accordance with the Swedish Public Access to Information and Secrecy Act, but can be made available to researchers upon request (subject to a review of secrecy).

## Supplementary information

**Supplementary information** The online version contains supplementary material available at 10.1038/s41533-021-00218-y.
