# Supplementary material for: Critical inhaler technique errors in Swedish patients with COPD: a cross-sectional study analysing video-recorded demonstrations
Source: NPJ Prim Care Respir Med. 2021 Feb 9;31:5. doi: 10.1038/s41533-021-00218-y (PMC7873256; doi:10.1038/s41533-021-00218-y)
Supplement: Supplementary file 1 — Supplemental material [file 41533_2021_218_MOESM1_ESM.pdf]

Supplementary information

Critical inhaler technique errors in Swedish patients with COPD – a cross-sectional study analysing video-recorded demonstrations

Johanna Sulku, Kristina Bröms, Marieann Högman, Christer Janson, Karin Lisspers, Andrei Malinovschi, Håkan Melhus, Björn Ställberg, Elisabet I Nielsen

Contents

Supplementary Table 1. Checklist for the Breezhaler DPI and the Handihaler DPI........2

Supplementary Table 2. Checklist for the Diskus DPI........3

Supplementary Table 3. Checklist for the Easyhaler DPI........3

Supplementary Table 4. Checklist for the Genuair DPI and the Novolizer DPI........4

Supplementary Table 5. Checklist for the Turbuhaler DPI........4

Supplementary Table 6. Checklist for the Respimat SMI........5

Supplementary Table 7. Inhaler device model combinations used by the patients (n = 364) included in the assessment of critical errors........6

Supplementary Table 8. Unadjusted odds ratios (ORs) with 95% CIs for association of variables with ≥ 1 critical error (CE) for at least one inhaler device model in an unadjusted simple logistic regression analysis........7

Supplementary Table 1. Checklist for the Breezhaler DPI and the Handihaler DPI

☐ Genuair

☐ Novolizer

	Inhaler technique checklist	Agree	Dis-agree	Not visible	Notes
1[1]	The patient opens the device.				
2[1]	The patient opens the mouthpiece, inserts the capsule into the chamber, and closes the mouthpiece. The device is held with the mouthpiece upwards.<br>☐ Breezhaler: The patient presses both side buttons simultaneously and then releases them.<br>☐ Handihaler: The patient presses the side button and releases it. The side button(s) is/are pressed only once and not pressed again at any time.				
3	The patient exhales distinctly before inhaling the dose.				
4[1]	The patient does not at any time exhale into the mouthpiece.				
5[1]	The patient places the mouthpiece in their mouth and breathes in through the mouth.				
6[1]	☐ Breezhaler: The patient breathes in slowly, steadily, and deeply, and the spinning sound can be heard.<br>☐ Handihaler: The patient breathes in slowly and deeply, and the spinning sound can be heard.				
7	The patient distinctly holds his/her breath after inhalation.				
8	The patient resumes normal breathing after inhaling the dose.				
9	The patient removes the empty capsule.				
10	The patient closes the device.				

[1]a critical error

Supplementary Table 2. Checklist for the Diskus DPI

	Inhaler technique checklist	Agree	Dis-agree	Not visible	Notes
1[1]	The patient opens the device and a click can be heard.				
2[1]	The patient holds the device with the mouthpiece toward him-/herself and slides the lever only once, until a click can be heard. The patient does not shake or turn the inhaler device upside down after sliding the lever.				
3	The patient exhales distinctly before inhaling the dose.				
4[1]	The patient does not at any time exhale into the mouthpiece.				
5[1]	The patient places the mouthpiece in his/her mouth and breathes in through the mouth.				
6[1]	The patients breathes in steadily and deeply.				
7	The patient distinctly holds his/her breath after inhalation.				
8	The patient resumes normal breathing after inhaling the dose.				
9	The patient closes the device and a click can be heard.				

[1]a critical error

Supplementary Table 3. Checklist for the Easyhaler DPI

	Inhaler technique checklist	Agree	Dis-agree	Not visible	Notes
1[1]	The patient opens the protective cover (if using it) and the device.				
2[1]	The patient shakes the device forcefully up and down more than 3 times, and holds it in the upright position. The device is not shaken again at any time after this. The patient presses the inhaler between their finger and thumb until a click can be heard, and then lets it click back. The button is pressed only once and the device is held upright after the button is pushed.				
3	The patient exhales distinctly before inhaling the dose.				
4[1]	The patient does not at any time exhale into the mouthpiece.				
5[1]	The patient places the mouthpiece in his/her mouth and breathes in through the mouth.				
6[1]	The patient breaths in strongly and deeply.				
7	The patient distinctly holds his/her breath after inhalation.				
8	The patient resumes normal breathing after inhaling the dose.				
9	The patient closes the device and the protective cover (if using it).				

[1]a critical error

Supplementary Table 4. Checklist for the Genuair DPI and the Novolizer DPI

☐ Genuair
☐ Novolizer

	Inhaler technique checklist	Agree	Dis-agree	Not visible	Notes
1[1]	The patient opens the device.				
2[1]	The patient holds the inhaler horizontally with the mouthpiece facing him-/herself and the coloured button on top. The patient presses the coloured button and a click can be heard, and then releases the button and does not push it again at any time.				
3	The patient exhales distinctly before inhaling the dose.				
4[1]	The patient does not at any time exhale into the mouthpiece.				
5[1]	The patient places the mouthpiece in his/her mouth and breathes in through the mouth.				
6[1]	The patient inhales strongly and deeply, and a click can be heard.				
7	The patient distinctly holds his/her breath after inhalation.				
8	The patient resumes normal breathing after inhaling the dose.				
9	The patient closes the device.				

[1]a critical error

Supplementary Table 5. Checklist for the Turbuhaler DPI

	Inhaler technique checklist	Agree	Dis-agree	Not visible	Notes
1[1]	The patient opens the device.				
2[1]	The patient holds the device upright with the coloured grip at the bottom, turns the coloured grip as far as it will go in one direction, and then turns it back so that a click can be heard. This is only done once.				
3	The patient exhales distinctly before inhaling the dose.				
4[1]	The patient does not at any time exhale into the mouthpiece.				
5[1]	The patient places the mouthpiece in his/her mouth and breathes in through the mouth.				
6[1]	The patient breathes in deeply and strongly.				
7	The patient resumes normal breathing after inhaling the dose.				
8	The patient closes the device.				

[1]a critical error

Supplementary Table 6. Checklist for the Respimat SMI

	Inhaler technique checklist	Agree	Dis-agree	Not visible	Notes
1[1]	The patient keeps the cap closed. The patient turns the clear base in the direction of the arrows on the label until a click can be heard (half a turn).				
2[1]	The patient opens the device.				
3	The patient exhales distinctly before inhaling the dose.				
4[1]	The patient places the mouthpiece in his/her mouth, pointing it toward the back of the throat, and breathes in through the mouth.				
5[1]	The patient takes a slow, deep breath and simultaneously presses the dose-release button, continuing to breathe in slowly for as long as is comfortable.				
6	The patient distinctly holds his/her breath after inhalation.				
7	The patient resumes normal breathing after inhaling the dose.				
8	The patient closes the device.				

[1]a critical error

Supplementary Table 7. Inhaler device model combinations used by the patients (n = 364) included in the assessment of critical errors.

Inhaler device model combination	n (%)	Missing recordings (n patients)
One inhaler device model	91 (25%)	
Turbuhaler	33 (36%)	-
Breezhaler or Handihaler	22 (24%)	-
Easyhaler	15 (17%)	-
Genuair or Novolizer	12 (13%)	-
Respimat	6 (7%)	-
Diskus (Accuhaler)	3 (3%)	-
Combination of two device models	199 (55%)	
Handihaler + Turbuhaler	90 (45%)	Handihaler (2), Turbuhaler (2)
Genuair or Novolizer + Turbuhaler	24 (12%)	-
Respimat + Turbuhaler	16 (8%)	-
Easyhaler + Handihaler	15 (8%)	Handihaler (1)
Easyhaler + Respimat	13 (7%)	Easyhaler (1)
Easyhaler + Genuair or Novolizer	9 (5%)	-
Easyhaler + Turbuhaler	9 (5%)	Turbuhaler (1)
Diskus (Accuhaler) + Handihaler	7 (4%)	-
Breezhaler or Handihaler + Genuair or Novolizer	6 (3%)	-
Diskus (Accuhaler) + Respimat	3 (2%)	-
Diskus (Accuhaler) + Turbuhaler	1 (0.5%)	-
Diskus (Accuhaler) + Novolizer	1 (0.5%)	-
Easyhaler + *pMDI suspension*	1 (0.5%)	-
Genuair + *Spiromax*	1 (0.5%)	-
Handihaler + *Spiromax*	1 (0.5%)	-
Novolizer + Respimat	1 (0.5%)	-
Spiromax + Turbuhaler	1 (0.5%)	-
Combination of three device models	69 (19%)	
Diskus (Accuhaler) + Handihaler + Turbuhaler	17 (25%)	Diskus (1), Turbuhaler (2)
Easyhaler + Handihaler + Turbuhaler	11 (16%)	Easyhaler (1), Turbuhaler (1)
Diskus (Accuhaler) + Respimat + Turbuhaler	4 (6%)	Diskus (1), Diskus + Turbuhaler (1), Turbuhaler (1)
Easyhaler + Genuair or Novolizer + Turbuhaler	4 (6%)	Easyhaler (1), Turbuhaler (1)
Easyhaler + Respimat + Turbuhaler	4 (6%)	Easyhaler (1)
Handihaler + Turbuhaler + *pMDI suspension*	4 (6%)	Turbuhaler (1)
Handihaler + Turbuhaler + *nebuliser*	3 (4%)	-
Breezhaler/Handihaler + Turbuhaler + *pMDI solution*	2 (3%)	-
Diskus (Accuhaler) + Genuair + Turbuhaler	2 (3%)	-
Diskus (Accuhaler) + Handihaler + *pMDI suspension*	2 (3%)	Diskus (1)
Diskus + Turbuhaler + *nebuliser*	2 (3%)	-
Easyhaler + Handihaler + Novolizer	2 (3%)	-
Handihaler + Turbuhaler + *pMDI breath-actuated*	2 (3%)	-
Diskus (Accuhaler) + Easyhaler + *nebuliser*	1 (1%)	Diskus (1)
Diskus (Accuhaler) + Easyhaler + Respimat	1 (1%)	-
Diskus (Accuhaler) + *Ellipta* + Handihaler	1 (1%)	-
Diskus (Accuhaler) + Genuair + *pMDI suspension*	1 (1%)	-
Ellipta + Genuair + Turbuhaler	1 (1%)	-
Genuair + *Spiromax* + Turbuhaler	1 (1%)	-
Handihaler + *pMDI suspension* + *nebuliser*	1 (1%)	-
Handihaler + Novolizer + Turbuhaler	1 (1%)	-
Handihaler + *Spiromax* + Turbuhaler	1 (1%)	-
Respimat + Turbuhaler + *nebuliser*	1 (1%)	-
Combination of four device models	5 (1%)	

Breezhaler + Easyhaler + *pMDI breath-actuated* + *nebuliser*	1 (20%)	-
Diskus (Accuhaler) + Easyhaler + Handihaler + Turbuhaler	1 (20%)	-
Easyhaler + Handihaler + Turbuhaler + *nebuliser*	1 (20%)	-
Easyhaler + Respimat + Turbuhaler + *pMDI suspension*	1 (20%)	-
Novolizer + Respimat + *Spiromax* + *nebuliser*	1 (20%)	-

Inhaler device models not included in the assessment of critical errors are shown in *italics*.

Supplementary Table 8. Unadjusted odds ratios (ORs) with 95% CIs for association of variables with ≥ 1 critical error (CE) for at least one inhaler device model in an unadjusted simple logistic regression analysis

Variable	n All n = 364	n CEs n = 242	OR (95% CI)	P value[1]
Sex				
Female	212	145	1.23 (0.78, 1.90)	0.36
Male	152	97	1	
Age, years				
< 65	84	52	1	
65–75	203	135	1.22 (0.72, 2.07)	0.46
> 75	77	55	1.54 (0.80, 3.04)	0.20
BMI, kg/m^2				
< 22	48	29	0.79 (0.42, 1.52)	0.47
22–30	217	143	1	
≥ 30	96	67	1.20 (0.72, 2.03)	0.50
Current smoker	98	69	1.34 (0.82, 2.25)	0.26
Education				
Elementary school	224	148	1	
Upper secondary school	95	64	1.06 (0.64, 1.78)	0.82
University	44	30	1.10 (0.56, 2.25)	0.79
Lung function				
$FEV_1 \geq 80\%$	24	14	1	
$50\% \leq FEV_1 < 80\%$	198	132	1.43 (0.59, 3.37)	0.42
$30\% \leq FEV_1 < 50\%$	105	66	1.21 (0.48, 2.97)	0.68
$FEV_1 < 30\%$	37	30	3.06 (0.98, 10.1)	0.06
CAT score ≥ 10	227	158	1.44 (0.92, 2.26)	0.11
mMRC score ≥ 2	172	117	1.14 (0.74, 1.77)	0.56
Frequent exacerbations	76	60	2.18 (1.22, 4.10)	0.01
Care level				
Primary care<br>Secondary care	324<br>40	208<br>34	1<br>3.16 (1.38, 8.56)	0.01
Planned visits to physician	150	108	1.56 (1.00, 2.46)	0.05
Planned visits to asthma/COPD nurse	255	168	0.94 (0.58, 1.51)	0.80
Number of device models used				
1 device model<br>2 device models<br>≥ 3 device models	91<br>199<br>74	39<br>139<br>64	1<br>3.09 (1.85, 5.19)<br>8.53 (4.03, 19.6)	 < 0.0001<br>< 0.0001

Data presented as number (%) of patients, BMI body mass index, CAT COPD assessment test, COPD chronic obstructive pulmonary disease, CI confidence interval, FEV_1 forced expiratory volume in 1 second, Frequent exacerbations ≥ 2 exacerbations treated in primary care or at emergency department and/or ≥ 1 hospital admissions during the previous year due to worsening in COPD, mMRC Modified Medical Research Council Dyspnea Scale.

[1]Comparison of variables between the subgroups were tested with Pearson's $\chi 2$ test for categorical variables and with unpaired t-test for continuous variables.
